# Interrelationship between Climatic, Ecologic, Social, and Cultural Determinants Affecting Dengue Emergence and Transmission in Puerto Rico and Their Implications for Zika Response

**DOI:** 10.1155/2017/8947067

**Published:** 2017-06-22

**Authors:** Angela Matysiak, Amira Roess

**Affiliations:** Department of Global Health, Milken Institute School of Public Health, The George Washington University, Washington, DC, USA

## Abstract

**Objective:**

The global resurgence of dengue has been attributed to rapid population growth, urban expansion, increased air travel, globalization, and climate change. Dengue is now endemic in Puerto Rico. Puerto Rico is at risk for Zika, another emerging arbovirus. The interrelationship between climatic, ecological, social, and cultural factors that affect dengue and other arboviruses' transmission is understudied.

**Design:**

The objective of this systematic review is to examine the interrelationship between climatic, ecological, social, and cultural factors on dengue transmission in Puerto Rico and to draw lessons for Zika response.

**Results:**

A comprehensive search of peer-reviewed journal articles was performed, producing 562 articles; 26 were selected for this review. Findings indicate that human activities and behaviors (urbanization, migration, and consumption) as well as climate have a significant impact on the abundance and the transmission potential of* Ae. aegypti*, the vector for dengue, Zika, and other viruses.

**Conclusion:**

Despite the public health burden of dengue limited investments have been made in research and surveillance. Future research is needed to develop models that integrate the multivariate effects of climatic, ecological, social, and cultural factors, which for Puerto Rico have mostly been examined independently. Such models have the potential to inform response to dengue, Zika, and other arboviruses.

## 1. Introduction

The recent Zika virus epidemic in Latin America highlights the need to study the ecology of arboviruses and apply lessons learned to the control of these diseases and their vectors [1]. Puerto Rico is at risk for an explosive Zika virus epidemic. We review the ecology of dengue in Puerto Rico and draw parallels between dengue and Zika.

Dengue is the most common and fastest-growing vector-borne viral disease in the world today and a significant global health problem with an estimated 50–100 million infections worldwide. There are approximately 500,000 cases of dengue hemorrhagic fever (DHF) and 22,000 deaths annually, mainly among children under the age of five [2, 3]. DHF fatality rates exceed 20% without proper treatment [4]. An estimated 2.5 billion people (40% of the world's population) in over 100 countries are at risk of infection, and approximately 975 million of these live in urban areas in tropical and subtropical countries in the Americas, Southeast Asia, and the Western Pacific [5]. Dengue is classified as a Neglected Tropical Disease (NTD) that disproportionally affects low-income and underprivileged populations in developing regions with a low standard of living [4]. Thus dengue is also known as a “Disease of Poverty” [6]. Dengue Fever (DF) and DHF (its more severe from) have been reemerging and expanding globally over the past 50 years at alarming rates [2, 3].

The principle vector for dengue virus (DENV) is the mosquito* Aedes aegypti*, which is found in many tropical and semitropical areas of the world, primarily in urban areas. The mosquito vector feeds on blood and breeds primarily in stagnant water like that found in discarded containers and car tires [2, 7]. The virus is transmitted to humans through the bites of infected female mosquitoes [2]. The mosquito has four distinct stages in its life cycle: egg, larva, pupa, and adult. The duration of the larval stages is 7–9 days at 25°C and that of the pupal stages is 2-3 days at the same temperature [8]. After feeding on an infected host, the transmission of virus can occur after an 8–10-day extrinsic incubation period (EIP) [2, 3].

Higher temperatures can increase the rate of larval development and the subsequent emergence of adult vectors as well as the vector-biting rate [2]. With a 2°C increase in temperature the EIP of DENV will be shortened, which will then cause vectors to bite more frequently [9]. All four serotypes produce a similar illness (characterized by fever, intense headache, rash, nausea, vomiting, and muscles and joint pains) and introduce life-long immunity that is specific to the infecting serotype. Dengue infections can also lead to DHF and excessive capillary permeability that can lead to dengue shock syndrome (DSS) and death [4, 8]. The risk of developing DHF appears to increase with a secondary infection with a different serotype [2]. Individuals experiencing a second infection (e.g., DEN-3 followed by DEN-1) are at least 15 times more likely to contract DHF/DSS [10].

Dengue epidemics inflict a significant health, economic, and social burden on the populations of endemic areas [11, 12]. In endemic areas in Asia and the Americas, the burden of dengue is approximately 1,300 disability adjusted life years (DALYs) per million population [11]. In 2001, the Americas alone reported over 652,212 cases of dengue of which 15,500 were DHF, nearly double the cases reported for the same region in 1995 [12]. By 2007, the annual incidence rate there reached nearly 900,000 cases, with more than 25,000 persons afflicted with DHF [6]. In 2010, 1.6 million cases were reported of which 49,000 were severe [13]. In 2011, more than one million cases were reported to the Pan American Health Organization (PAHO), including 18,070 cases of DHF and/or DSS. There has been a dramatic increase in the number of reported cases in the region, including Brazil, Paraguay, the Bahamas, Aruba, and St. Lucia. Brazil had three times the rate in 2011 (with 388 per 100,000 population) as in 2005 (with 118 per 100,000 population). Dengue is endemic in Puerto Rico (where it has caused epidemics since the 1960s) [14].

Puerto Rico (PR) has experienced increasingly severe epidemics since the introduction of multiple dengue serotypes beginning in the 1970s with a pattern of epidemics in 2-3 year cycles for each of the prevailing serotypes [15]. Since the early 1980s PR has experienced continued hyperendemic dengue transmission, which is demonstrated by the occurrence of multiple cocirculating serotypes at a given time. DEN-4 epidemics in 1986 and 1989 were followed by the emergence of DHF in 1986 and high incidences of DHF and DSS in subsequent years [15]. Dengue cases have been reported during each month of the year since 1986, indicating the endemic nature of dengue in Puerto Rico [16]. In nonepidemic years, reported cases of dengue range from approximately 3,400 to 7,000 per year [17]. In Puerto Rico, dengue is seasonal, with a large peak of cases reported during the rainy season, which typically runs from August to November [14].

**Figure 1:**
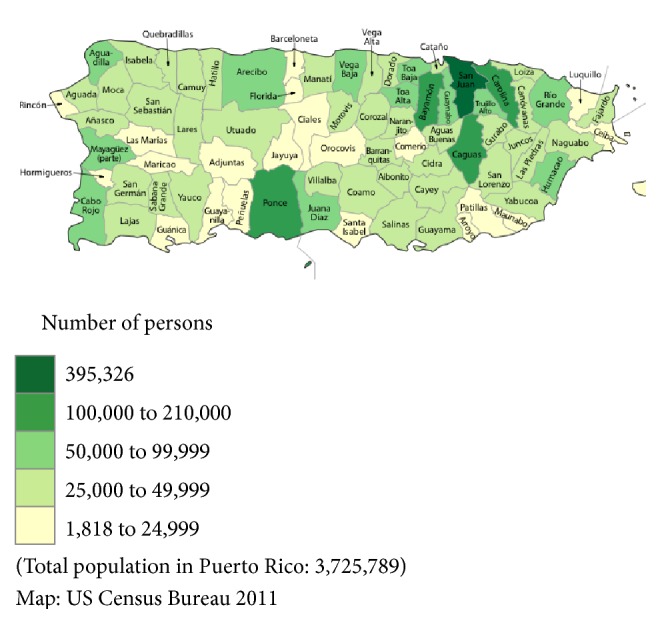
Puerto Rico population per municipality in 2010.

In 2010, Puerto Rico experienced the largest outbreak of dengue in its history, recording over 21,000 cases. Dengue incidence was highest in the densely populated northwestern region (see Figure 1 for map of PR). The incidence rate for 2010 was twice that of 2007, when over 10,500 cases were reported [18]. The disease surveillance system in Puerto Rico comprises primarily passive and some active surveillance activities [14]. The Dengue Branch of the CDC is located in San Juan and is responsible for the island-wide laboratory-based passive dengue surveillance system (PDSS) in collaboration with the Puerto Rico Department of Health (PRDH) [18]. The active surveillance system includes diagnostic testing and submission of serum samples accompanied by a Dengue Case Investigation Report (DCIR) from suspected dengue case-patients to the CDC Dengue Branch Laboratories [18]. DF and DHF reporting is mandated by law in Puerto Rico [18].

### 1.1. Key Risk Factors Associated with Dengue

The global resurgence of dengue in endemic areas has been attributed to rapid population growth and urban expansion [3]. Unplanned urban expansion leads to high densities of humans exposed to the mosquito vector* Ae. aegypti* [3]. It also puts severe constraints on civic amenities, particularly water supply and solid waste disposal, thereby increasing the breeding potential of the vector species [4, 9]. Water that is left stored in the open can become a breeding ground for mosquitoes that carry dengue fever [19]. Risk factors associated with dengue also include lacking mosquito control infrastructure and dengue vector control services [20]. In addition, increased air travel and globalization of trade significantly contributed to the introduction of all dengue virus serotypes to most population centers of the world. Globalization and travel have also contributed to hyperendemic transmission of all four dengue serotypes throughout the tropics [21].

Changing dengue incidence and distribution have also been attributed to climate [22, 23]. Rainfall and temperatures affect the spread of mosquito vectors and virus transmission. Rising temperatures and changing rainfall patterns, two examples of climate change, have been linked to the expansion of the flight ranges of mosquitoes carrying malaria* (Anopheles)*, dengue fever* (Ae. Aegypti),* and other tropical diseases [24]. Climate change has been associated with increases in dengue incidence and distribution in Puerto Rico, Mexico, and Thailand [25].

Indeed, global warming is predicted to lead to an increase in global temperatures between 2 and 4.5 degrees' Celsius by the year 2100 and could have a perceptible impact on vector-borne diseases. Climate change is projected to lead to a substantial increase in populations at risk of dengue and could expose an additional two billion people to dengue transmission by 2080 [26]. Studies also predict that global climate change could affect sylvatic dengue virus (DENV) affecting wild animals, as compared to urban DENV circulation, and lead to cross-species transmission of DENV into humans [27]. It is also conjectured that climate change and climate variability have the greatest adverse effect on small island states [28–30].

### 1.2. Zika Virus

Zika virus (ZIKV), an emerging vector-borne* Flavivirus*, was initially isolated from a rhesus monkey in the Zika forest in Uganda in 1947 [31]. The classic clinical picture of ZIKV infection resembles that of other arboviruses found in the locations where dengue occurs including dengue fever and chikungunya. Symptoms include fever, headache, arthralgia, myalgia, and maculopapular rash; thus differential diagnosis is difficult [31].

ZIKV is transmitted primarily by* Aedes aegypti* and* Aedes albopictus* and sexually transmitted cases have been reported [32]. Infection can be asymptomatic, can result in acute febrile illness with rash [33], and is associated with Guillain-Barré syndrome (GBS) [34] and severe thrombocytopenia [35, 36]. Infection during pregnancy can lead to microcephaly and other severe birth defects [37]. Sporadic cases have been reported in Southeast Asia and sub-Saharan Africa [38] with major epidemics in French Polynesia, New Caledonia, the Cook Islands, and Easter Island in 2013 and 2014 [39, 40]. In 2015, there was a dramatic increase in reports of ZIKV infection in the Americas. Brazil is the most affected country, with preliminary estimates up to 1.3 million cases [41]. In December 2015, the Puerto Rico Department of Health (PRDH) reported the first locally acquired case of Zika virus infection and as of July 29, 2016, 5572 cases have been confirmed [41].

### 1.3. Community Profile Puerto Rico

#### 1.3.1. Ecosystem and Climate

Located in the Caribbean Sea 950 miles southeast of Miami, Florida, Puerto Rico is an archipelago comprised of six islands with a land area of 3,421 square miles. The main island is 110 miles long with a mountainous central area surrounded by sandy beaches and coral reef along almost the entire coastal area. PR is characterized by a tropical climate with an average annual temperature of 25°C. The dry season typically lasts from December to April and the wet season (hot and rainy) runs from May to November. Hurricanes, high rainfall, landslides, and droughts have been major natural disturbances on the island. A former Spanish colony, Puerto Rico was acquired by the United States in 1898 and became an incorporated US territory in 1917 [42].

#### 1.3.2. Population Demographics

Puerto Rico is the fourth largest island after Cuba, Hispaniola (Haiti and the Dominican Republic), and Jamaica in the Greater Antilles. Puerto Rico's population has increased over the last four decades by almost 40% from 2,712,033 in 1970 to 3,725,789 in 2010 [43]. Puerto Rico experienced rapid urbanization (living in towns of 2,000 or more) from 40.5% in 1900, to 71% in 1950 and 100% of the total population living in urban areas today [44]. Puerto Rico is completely urbanized. Puerto Rico's population density has quadrupled over the last century from 107 persons per km^2^ in 1898 to 450 persons per km^2^ in 2011 making it one of the most densely populated areas in the region [45].

#### 1.3.3. Social and Economic Factors

In 2009, 25.4% of the adult population (25 years of age or over) had a high school diploma. Approximately 13.0% had some college, 8.6% held an associate's degree, and 16.1% had a bachelor's degree. These rates are below the US, where 85.3% of the adult population had a high school degree, 27.0% a bachelor's degree or more, and 10.3% an advanced degree. In 2009, 39.5% of the civilian population 16 years of age and over was employed and most work for Educational and Social Services (21.5%) followed by Retail Trade (11.5%) and Manufacturing (11.2%) [43]. The recent economic crisis has resulted in an increase in immigration into the United States [46].

## 2. Purpose and Objective

While there is ample research to suggest that climate change has a serious impact on disease emergence, though in some cases this research is contradictory, there is, however, little research that examines the relationship between climate and environmental and/or ecological conditions. This systematic literature review synthesizes and presents the status of current research on the effects of the interrelationship between climatic, ecological, social, and cultural factors on dengue emergence and transmission [26] in order to inform future dengue and Zika disease prevention and control strategies in Puerto Rico.

For the purpose of this synthesis, environmental factors are those external to the individual human host and include economic and social factors and ecological factors are those pertaining to the biology and ecology of the mosquito vector. Climatic factors are variations in temperature and precipitation that may influence the biology and ecology of mosquito vectors. This paper will not address intrinsic factors associated with the host (such as those pertaining to human immunity) and the agent (the dengue* Flaviviridae* virus). Puerto Rico was chosen as a case study for this project because of the endemic and epidemic nature of dengue fever on the island, the availability of a rich body of research literature, and the growing Zika virus epidemic.

## 3. Methodology

### 3.1. Identification of Studies

**Figure 2:**
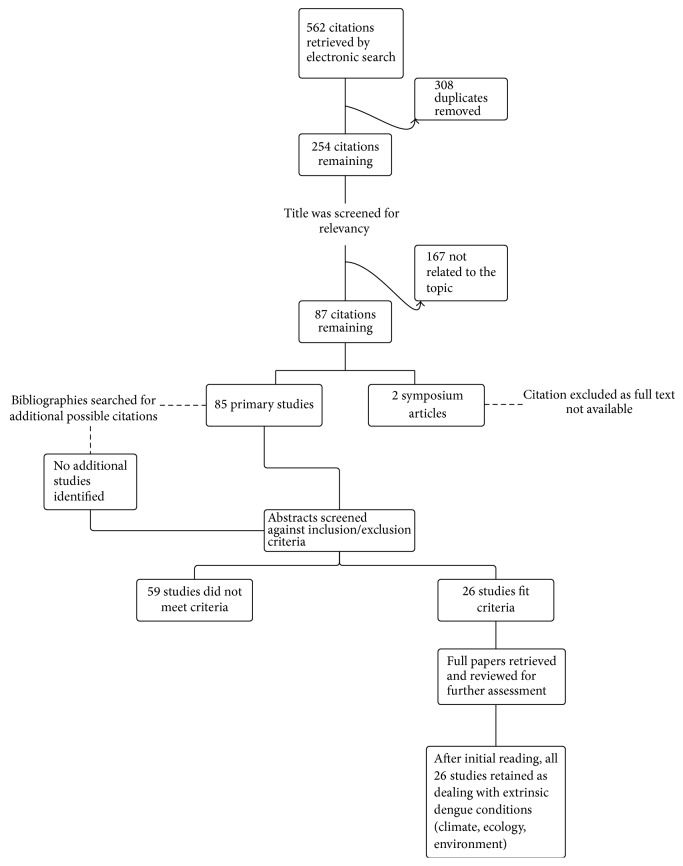
Literature selection and review process.

A keyword combination search was made for peer-reviewed journals published between 2001 and 2015 in English using MEDLINE, PUBMED, SCOPUS, and CINAHL. Keyword combinations were “dengue” AND “Puerto Rico” (OR “climate” OR “climate change” OR “seasonal variation” OR “hurricane” OR “rainfall” OR “precipitation” OR “moisture” OR “population density” OR “population growth” OR “migration” OR “travel” OR “housing” OR “water” OR “sanitation” OR “water storage” OR “costs” OR “behavior” OR “knowledge” OR “control” OR “service” OR “prevention” OR “environment” OR “ecology” OR “ecological expansion”). This process was complemented by searching in Google Scholar and supplemented with additional information from the CDC, PAHO, and WHO. Article's bibliographies were manually searched for additional citations (see Figure 2 for search results).

### 3.2. Inclusion and Exclusion Criteria

All studies retrieved from databases were evaluated for inclusion in the systematic literature review based on a combination of preselected criteria and whether or not they pertained to the research questions. The following inclusion criteria were used: (1) peer-reviewed studies from all disciplines that used qualitative and/or quantitative methods (mixed methods) and that reported qualitative or quantitative findings; (2) studies that were published in English between 2001 and 2015; and (3) unpublished and published studies that analyzed or described the role of extrinsic dengue transmission factors in Puerto Rico. The following exclusion criteria were used: (1) studies that provided no analysis or description of political, social, and economic conditions, ecology or climate; (2) studies that considered intrinsic factors pertaining to the virus and the host (e.g., studies on molecular biology, vaccine development, and clinical studies); and (3) articles that addressed West Nile Virus (WNV), yellow fever, or other vector-borne viruses in the Caribbean or Latin America.

Grey literature or other documents not peer-reviewed were excluded because only rigorous research was sought.

All studies were conducted in Puerto Rico and published between 2001 and 2015; one study was a multicountry perspective, which included Thailand, in addition to Puerto Rico [47]. The articles were comprised of 21 quantitative studies, 2 mixed methods, and 3 qualitative (see Appendix  A in Supplementary Material available online at https://doi.org/10.1155/2017/8947067). Most of the included studies investigated only one or two variables. For example, three studies investigated temperature, precipitation, and dengue incidence. One study considered temperature and dengue incidence. Two studies investigated larval and pupal abundance and productivity and water temperature. Three studies considered the relationship between subterranean aquatic habitats (septic tanks) and* Ae. aegypti* productivity. (A summary table of social, cultural, climatic, and ecological research on dengue in Puerto Rico by dengue transmission factor/variables can be found in Appendix  B.)

### 3.3. Study Analysis

The search identified 562 articles. After eliminating the duplicates, article titles were screened for relevancy and 87 citations remained. After scanning all abstracts against inclusion and exclusion criteria, 26 articles remained, 11 of which were journal articles that considered social and cultural transmission factors, and 7 papers that referenced ecological transmission factors. Additionally, 8 studies about climate were included. See Figure 2 for an overview of the selection and review process.

### 3.4. Quality Assessment Method

All studies that met the eligibility criteria were subsequently analyzed for relevant data. Data was extracted from key results, discussion, and conclusions. All studies were evaluated and rated based on the following criteria adapted from Fink 2010 [48]: (1) design and sampling method; (2) reliability and validity of data collection; (3) program or intervention; (4) data analysis; (5) results; and (6) conclusions. Studies were reviewed for whether it satisfied each of Fink's criteria, examined in the form of 49 questions. Each question was answered Yes, No, or Not Applicable (NA). A Yes answer received 1 point, a No answer received zero points, and criteria that were Not Applicable because of the study design were disregarded. Each study was then given an overall quality score represented by the quintile range of the percentage of applicable Fink criteria answered in the affirmative. If the study satisfied 80–100% of applicable Fink criteria, it received a quality score of 5, satisfying between 60 and 79% resulting in a score of 4, those between 40 and 59% received a score of 3, those between 20 and 39% received a score of 2, and those between 0 and 19% received a score of 1. A score of 1-2 was considered fair; scores between 3 and 4 were deemed moderate; and a score of 5 was considered strong. (A quality assessment checklist with 49 questions can be found in Appendix  C.)

## 4. Results

The results of this literature review are divided into three parts. Part one assesses the quality of the reviewed articles. Part two lists the effects of climatic, ecological, and social, and cultural conditions on the transmission of dengue in Puerto Rico. Part three presents the interrelationship between nonclimatic and climate factors in the transmission of dengue in Puerto Rico.

### 4.1. Part 1: Quality Assessment

Article quality was assessed to describe the research and not to screen out any of the remaining 26 articles.

Ten studies (38.5%) satisfied 80% or more of the applicable criteria (range 80%–93%), achieving a score of five and a “high” rating (see Table 1). Though none of the 26 studies satisfied 100% of applicable Fink criteria, all studies analyzed satisfied at least 33% of the relevant Fink criteria, achieving a score of two or more, and were therefore deemed of “moderate” quality. Higher quality studies addressed their limitations, in design or sampling methodology or related impact. (see Appendix  C for scoring criteria; see Appendix  D for a detailed list of quality rating results).

### 4.2. Part 2: Effects of Climatic, Ecological, and Social, and Cultural Conditions on Dengue Transmission (See Table 2 for a Summary of Results)

#### 4.2.1. Studies on Climate

Six studies examined the association between climate and dengue transmission [22, 25, 48–51]. Johansson et al. (2009), Jury (2008), and Méndez-Lázaro et al. (2014) explored the relationship between temperature, precipitation, and dengue incidence [25, 48, 50]. Johansson et al. (2009) found a positive association between short-term interannual (monthly) variations in temperature, precipitation, and dengue incidence [25]. This positive association varied spatially and was associated with differences in local climate. For example, the effect of temperature on dengue incidence was highest in Puerto Rico's cool and wet mountainous area, while the effect of precipitation was greatest in the hot and dry southwestern coastal region. Likewise, Méndez-Lázaro et al. (2014) found that dengue transmission increased by a factor of 3.4 for each 1°C increase in sea surface temperature. In contrast, Jury (2008) found that monthly increases in dengue cases were driven by rainfall (May to November) while intra-annual variability in dengue cases related positively to temperature. Jury also found that the incidence of dengue had been relatively constant over time, despite global warming, which was possibly due to declining rainfall after 1998, improving health care and small changes in population size. He also found a weak association between year-to-year variability in dengue cases and local rainfall and an Index of El Nino Southern Oscillation (ENSO) [48]. Keating (2001) considered the relationship between temperature and dengue incidence. Keating (2001) found a positive association between short-term interannual (monthly) variations in temperature and dengue incidence (as the temperature increases so does the number of dengue cases reported each month in Puerto Rico) [49].

Two studies also considered the effect of interannual climate variations (ENSO) on dengue incidence [48, 51]. Jury found that intra-annual variability in dengue cases was only weakly associated with ENSO [48]. Rigau-Pérez et al. found no positive relationship between the increase in dengue transmission and hurricanes and floods in 1995 and 1996, respectively [51]. He also found no significant correlation between increased transmission and rationed water supply and closed local landfills. Another study predicted that declining rainfall, improving health care, and limited population growth offset adverse effects of global warming [48]. A consideration of the potential mediating or constraining influence of nonclimatic factors of dengue transmission (socioeconomic factors) by Johansson et al. (2009) revealed that the association between weather variables and dengue incidence was stronger in areas with a higher poverty index [25]. Keating (2001) found that factors other than climate might be contributing to seasonal dengue incidence in Puerto Rico, such as predator-prey relationships, herd immunity, demographic changes, precipitation, and socioeconomic fluctuations [49].

#### 4.2.2. Studies on Ecological Factors

Seven studies discussed the effects of ecological factors on vector breeding and/or pupal production [7, 16, 47, 52–55]. Four of these examined the ecology of surface containers. In a multivariate regression analysis, the number of pupae in yard containers showed a positive and significant association with the number of trees per household, water volume, and lower water temperature [53]. Factors associated with increased breeding and/or pupal productivity showed a consistent pattern across sites. The* Ae. aegypti* population was most abundant and reproduced most successfully in discarded and unattended yard containers located under shaded trees and with filled with rainfall that had passed through foliage [52, 53]. (Trees provide shade, lower evaporation rates, and protection against extreme water temperatures, factors that may likely determine* Ae. aegypti's* development and survival.)

One study explored the effect of landscape elements (forests, high-density housing, and low-density housing) on the distribution of containers with* Ae. aegypti* and* Ae. mediovittatus* in urban, suburban, and rural areas [7].* Ae. aegypti* showed a positive association with high-density housing, urban regions, and elevated water temperature in bamboo pots, whereas* Ae. mediovittatus* was positively associated with forest areas, rural regions, and negatively correlated with water temperature. This study confirmed that* Ae. aegypti* is well adapted to the environments of crowded tropical cities and to a variety of terrestrial environments with or without tree coverage and associated with humans [7]. Another study examined the effect of housing distribution and patterns on* Ae. aegypti* dispersal in rural communities and found that in rural habitats* Ae. aegypti* do not disperse far from their development sites and tend to be spatially clustered at the household level [47].

Of the seven studies discussing the effects of ecological factors on vector breeding and/or pupal production, three studies examined the relationship between subterranean aquatic habitats, septic tanks (ST), and* Ae. aegypti* productivity [16, 54, 55]. Barerra reported that, during the dry season, septic tanks produce significantly more* Ae. aegypti* than surface containers (8-9 pupae/person versus 1.3–2.0 pupae/person on average) [16]. It is important to note that this study was the first study of aquatic habitats producing dengue vectors anywhere in the Caribbean region or in Central and South America; the findings were unexpected because* Ae. aegypti* had been regarded as a “clean water” mosquito. Burke et al. (2010) also found a positive association between the number of mosquitoes and the conditions of septic tanks (cracked, uncapped openings, and septic water pH) in the dry season [54].

In expanding on Barrera's work, MacKay et al. (2009) found that septic tanks produced significantly more* Ae. aegypti* and* Cx. Quinquefasciatus*, the latter a vector for other arboviruses, throughout the year, and not just during the dry season. There was no significant relationship between temporal changes in mosquito production and rainfall. He also reported that* Ae. aegypti* adults emerging from septic tanks were significantly larger (3 to 9 times) than those emerging from surface water. This suggests that larval development in the nutrient-rich environment of septic tanks may provide a fitness advantage to adult* Ae. aegypti*. [55].

#### 4.2.3. Studies on Environmental Factors

Thirteen studies examined the effect of environmental factors on dengue emergence and disease severity [15, 17, 51, 56–65]. Of the articles discussing environmental factors, three considered entomologic surveillance and dengue vector control measures [58–60]. As mentioned earlier, pupal production is an indicator of vector abundance and density (vector density is the number of pupae per person). When vector density is considered high (all things being equal), the likelihood of an epidemic increases. Barrera et al. [59] found that sequential sampling provided substantial reductions in the sample size required to determine if vector density falls above or below a given threshold. For example, he found that, after sampling only 25 premises in the first survey and 125 in the second survey, it was possible to determine that pupae densities were above the dengue epidemic threshold (female pupae/person of 0.19) [59]. That study also examined the use of predictive computer models for validating threshold limits for epidemic risk in a particular locale [59]. He confirmed that simplified pupal surveys of* Ae. aegypti* (using sequential sampling programs) are an efficient method for validating entomological dengue transmission thresholds indicating when transmission might occur and thus forecasting epidemics [47]. Entomologic thresholds were based on mosquito simulation model and a dengue simulation model [59]. One study found that cost effective larval control programs are less than US$ 2.50 per person and reduce dengue transmission by 50%. Larval control programs in conjunction with an early warning system that provides information on possible outbreak of dengue are cost effective if below US$ 4.50 per person [60] (see Table 3).

Several articles highlighted the importance of Puerto Rico's surveillance system, which is maintained by PRDH and the Dengue Branch at the CDC. Rigau-Pérez et al. (2002) attributed the announcement in 1994 of a dengue epidemic (DEN-3) by the PRDH and the increase in dengue incidence in 1998 to the rapid implementation of education efforts for the general public and medical community [66]. Two studies evaluated the island's laboratory-based, enhanced dengue surveillance system (EDSS), which was developed by the CDC and first implemented in Puerto Rico in 2005 [63, 66]. Ramos et al. (2008) concluded that the island's enhanced surveillance system allowed for a more accurate, population-based estimate of dengue incidence and severity [64]. She also found that simplified case definitions for DHF based on WHO criteria might be useful for clinic-based surveillance. In addition, she postulated that early identification of dengue cases could be useful in reducing virus transmission in dengue endemic communities. Both Rigau-Pérez and Ramos stressed the importance of virologic surveillance in confirming the accuracy of dengue diagnosis [64, 66] (see Table 3).

*Population Growth*. One article addressed the effect of human population growth on epidemic dengue [15]. Bennett et al. found that increased human population density was positively associated with DEN-4 epidemics in Puerto Rico between 1981 and 1998 (and the subsequent emergence of epidemic DHF) [15] (see Table 3).

#### 4.2.4. Studies on Cultural and Behavioral Factors

Five studies considered cultural factors, such as community knowledge, attitudes, and practices (KAP), about dengue in Puerto Rico [17, 56, 61, 62, 65]. Two examined the impact of dengue intervention and control methods on dengue incidence [61, 65]. Winch et al. established that a pilot child-focused community program conducted in Puerto Rico in 1995 showed significant positive impact on knowledge and behavior related to dengue prevention [65]. For example, he identified that exposure to an exhibit on* Ae. aegypti* at the Children's Museum in San Juan and to elementary and preschool education programs was associated with significantly higher levels of correct dengue-related knowledge among children. He also ascertained a statistically significant increase in the proportion of protected tires with increasing parent-child communication about dengue [65]. Clark et al. confirmed that a child-focused social mobilization and communication program raised awareness and generated some behavior change [61]. Both authors reported that the pilot program had limited impact of larval indices [61, 65].

Two articles explored knowledge, attitudes, and practices (KAP) about dengue prevention in Puerto Rico's communities. Pérez-Guerra et al. found that while respondent's knowledge about dengue prevention was generally correct, major misconceptions related to attitudes and actions concerning dengue persisted in the general public [56]. The author identified several barriers to sustained dengue prevention: (1) misconceptions about dengue from outdated educational material; (2) the “invisibility” of dengue as compared to the importance of chronic diseases; (3) a lack of acceptance of responsibility for dengue prevention [17, 56]. Pérez-Guerra et al. also identified differences in KAP by gender, which were consistent with the cultural values and social norms in Puerto Rico. For example, women considered dengue important because of the burden and impact on society, while men thought it was a serious disease because of an individual's lack of perceived health risk [17]. One article inquired about the relationship between dengue education and personal protective measures against mosquitoes [62]. O'Leary et al. determined that pretravel educational messages contributed to protective behavior in travelers to endemic areas [62].

### 4.3. Part 3: The Interrelationship between Nonclimactic and Climate Factors in Dengue Transmission

Several studies considered the interactions between climate and ecological and environmental factors. Johansson et al. investigated the effect of temperature and precipitation across an elevational gradient (mountains and coastal areas) [25]. He found, as mentioned above, that the effect of temperature on dengue incidence was highest in Puerto Rico's cool and wet mountainous area, while the effect of precipitation was greatest in the hot and dry southwestern coastal region. Johansson also considered the potential mediating or constraining influence of socioeconomic factors of dengue transmission (population density, median household income, and the percentage of families living below the poverty line). He found that the association between weather variables and dengue incidence was stronger in areas with a higher poverty index [25]. And he also inferred that factors other than climate related to the history of herd immunity, the introduction of a new serotype, or demographic transitions are also influencing the cyclical transmission of dengue fever [49]. Jury (2008) reported that declining rainfall, improving health care, and limited population growth might offset adverse effects of global warming [48].

Cox investigated the effect of temperature across an urban to rural environmental and an elevation gradient [7]. The urban to rural gradient was associated with changes in land use and vegetation extents (due to human intervention) and with an elevation gradient from the coastal lowlands in the north of the island towards the northern foothills located in the South. He ascertained that* Ae. aegypti* was positively associated with water temperature, which reflected the greater exposure to the sun of bamboo pots in highly urbanized areas, as stated above.

As mentioned, climate variables such as temperature and precipitation influence the abundance and the transmission potential of* Ae. aegypti*. Three studies explored the interaction effects of climate (temperature and/or precipitation) and household level and human-related behavior (water storage practices and yard maintenance) on vector abundance and productivity [52, 53, 59]. Barrera et al. detected a positive association between containers with leaf litter or algae that had received rainfall through foliage and had lower water temperature (≤29°C) and larvae and pupae abundance [53]. Building on this work Morin et al. (2015) examined how the interactions among meteorological variables, mosquitoes, and the dengue virus influence transmission [22]. They concluded that current climatic conditions modify the influence of human and climatic factors on dengue. They report that, during drier years, containers filled with water by humans were the most important habitat for mosquitoes. In warmer years there was an increase in number of dengue cases that peaked following high rainfall.

**Figure 3:**
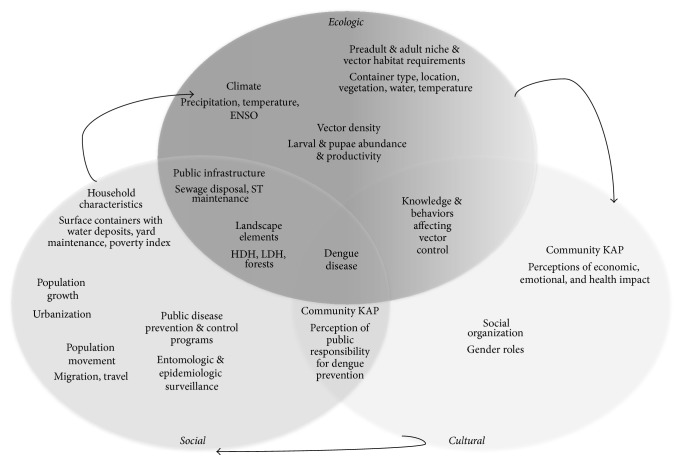
Study framework and results summarizing interrelationships between climatic, ecological, social, and cultural determinants affecting dengue emergence and transmission in Puerto Rico.

Overall, from this literature, 15 dengue transmission factors emerged: climate (temperature and rainfall), preadult and adult niche and vector habitat requirements, vector density, public infrastructure, landscape elements, household characteristics, population growth, population movement, public disease prevention and control programs, knowledge and behaviors affecting vector control, community knowledge, attitudes and behaviors related to dengue disease, and social organization. Within each factor, a range of variables was identified according to the emphasis provided in each article. The results of this review are presented in the study framework (Figure 3) adapted from Caprara et al. (2009) and illustrates the interrelationship of climatic, ecological, social, and cultural determinants affecting dengue emergence and transmission in Puerto Rico [67].

## 5. Discussion

This systematic literature review synthesized information on climatic, ecological, social, and cultural determinants affecting dengue emergence and transmission in Puerto Rico. Several key findings have implications for Zika emergence and control. First, increases in temperature and rainfall were associated with increased vector density and ultimately dengue transmission across Puerto Rico [25, 48]. Seasonal rainfall enhances mosquito breeding and therefore vector abundance in areas where outdoor rain-filled surface containers are being used [58] and discarded containers, those used for animal drinking, ornamental purposes, and household cleaning filled with standing water were the most common oviposition sites.* Ae. aegypti* is the main vector for Zika virus and it is likely that a similar seasonal pattern to dengue will be observed for Zika in PR.

Second, several studies provided insights in* Ae. aegypti* habitat and the potential for dengue risk in Puerto Rico [7, 16, 25, 47–55, 58]. The articles on this topic can be taken as an indicator that operational research on dengue ecology and epidemiology in Puerto Rico is strong and that applications of tested interventions to reduce dengue transmission can ultimately stop the rapid spread of Zika.

Third, septic tanks produced significantly more* Ae. aegypti* than surface containers throughout the year [16, 54, 55]. One study found that* Ae. aegypti* emerging from septic tanks were significantly larger (3 to 9 times) than those emerging from surface water. These findings suggested that larval development in nutrient-rich environment of septic tanks might provide a fitness advantage to adult* Ae. aegypti* [54]. The implications of these findings are significant as they suggest that the mosquito vector has adapted well to a multitude of urban habitats and environments and suggest that there may be other previously unknown breeding/oviposition sites.

The wealth of research on environmental factors (social and cultural) affecting dengue emergence and disease emergence in Puerto Rico reflects the relative strength of Puerto Rico's dengue prevention and control services. Two studies reported on the effectiveness of epidemiologic and entomologic disease surveillance programs administered under the auspices of the PRDOH and the CDC [64, 66]. One article demonstrated new strategies for dengue prevention and control and the benefits of new models and survey methods for the assessment of vector ecology to help target environmental management and control [58]. Further investment in these existing systems can slow down the spread of dengue, Zika, and other viruses.

Research on cultural factors, such as community knowledge, attitudes, and practices (KAP) about dengue in Puerto Rico showed important limitations: misconceptions about dengue due to a lack of outdated educational material; a lack of a perceived importance of dengue in the context of a growing concern over the impact of chronic diseases; a lack of personal responsibility for dengue prevention in the context of a decentralized public health system; and failure to communicate effectively across local cultures and genders [17, 56]. Several emerging diseases have faced the same communication problems including Ebola, MERS-CoV, and now Zika. Health communication campaigns are often poorly resourced, rarely engage health communication experts from the affected area, and seldom benefit from meaningful evaluation. Despite this, community-based interventions remain to be critical to achieve long-term vector control through behavior change [61, 65] and public health interventions should address existing barriers to behavior which are deeply entrenched in the local culture's belief about health as well as gender roles and promote the integration of community groups and community leaders into current dengue prevention and control programs [17, 56].

From this review it can also be shown that research and understanding on the effects of environmental and ecological transmission components of dengue are limited. The review also indicates that there are few studies on the interrelationships between climate and nonclimate transmission factors. Additional research in this area is needed to fully understand the dynamics of dengue transmission.

This systematic literature review yielded two important public health implications. First, although source reduction programs targeting the most productive surface container habitats remain an essential component of dengue prevention and control [61], interventions should address structural problems that increase mosquito populations. Infrastructure investments should be used to convert homes from open sewage (ST) to sewer use [16, 55, 56] and to provide access to safe and a reliable water supply [17]. Though substantial, such investments would have a major impact on vector ecology and reduce the health burdens caused by dengue, Zika, chikungunya, and other emerging viruses. In addition to health gains, these investments would also help alleviate some of the existing structural poverty on the island.

Second, though epidemiological data on human disease indicators are currently available to inform vector control services, this data is, to a large extent, through passive surveillance. Investments are needed to strengthen Puerto Rico's active surveillance system to achieve a higher quality of surveillance data which can be used to build and strengthen predictive models [58, 63, 64]. The incorporation of early warning data into larval control programs may also be beneficial, apart from the positive health effect, as it can improve cost-effectiveness of larval control programs [60].

There are several limitations in this literature review. First, this review was not exhaustive as only extrinsic transmission factors were included in the analysis. Other factors, such as intrinsic factors pertaining to human immunity and the dengue virus (infection parity, passively acquired antibodies, enhancing antibodies, absence of protective antibodies, viral strain, age, sex, race, nutritional status, and preceding host conditions), are important to consider in future studies. Second, there is an inherent risk of bias in the studies included in this review since only peer-reviewed studies were included (reporting bias). Third, there is also an inherit level of simplicity (relatively speaking) in the studies included as they investigated only one or two variables. Many of the included studies are limited by their design (ecological bias).

Nevertheless, this review indicates that extrinsic climatic, ecological, and environmental factors have an effect on dengue emergence and transmission in Puerto Rico. Changes in ecological factors, such as the vector utilizing alternative habitats such as septic tanks, and changes in human land use (urbanization) and vegetation provide new oviposition sites and feeding opportunities, allowing the vector to survive and increase its transmission potential. Social and cultural factors such as disease surveillance, community knowledge and behaviors (KAP), and social organization (gender role behaviors) play an important role in affecting vector control. These and other extrinsic factors do not act in isolation, but rather affect dengue transmission directly, indirectly, and in combination. Future research needs to further refine models in dengue transmission and include additional extrinsic factors such as demographic, geographic, and socioeconomic in order to understand further how they affect dengue transmission on a local level and over time. The problem of dengue, and now of Zika, transmission can change only if the vector can be controlled. However, for Puerto Rico, and in the context of global warming and increasing population growth and diversity, extrinsic factors are likely to remain influential well into the future.

## Supplementary Material

Appendix A: Summary of all studies included in the review. This table provides information about the study design, limitations, findings, and recommendations.
Appendix B: Summary table of study results grouped by the category of research (social, cultural, and/or ecological).
Appendix C: Quality assessment checklist with 49 questions developed and used in this review.
Appendix D: Results of the quality assessment. Each study was reviewed for whether it satisfied each of Fink's criteria, examined in the form of 49 questions. Depending on the study design applicable questions were answered with Yes, No, or Not Applicable (NA). A YES answer received 1 point, a NO answer received zero points and criteria that were not applicable because of the study design were disregarded. After all questions were answered for each study, the percentage of satisfied Fink criteria was calculated (total number of satisfied criteria divided by total number of applicable criteria). Each study was then given an overall quality score represented by the quintile range of the percentage of applicable Fink criteria answered in the affirmative.

## Figures and Tables

**Table 1:** Quality assessment result.

Satisfied criteria	Quality score	Quality rating	*n* (%)	Remarks
80–100%	5	Strong	10 (38.5)	Strong studies addressed sampling methodology, missing data, and their impact on the implications or conclusions drawn.
60–79%	4	Moderate	13 (50)	
40–59%	3	Moderate	2 (7.7)	
20–39%	2	Fair	1 (3.8)	
0–19%	1	Fair	0 (0)	

**Table 2:** Summary of results on the effects of climatic, ecological, and social and cultural conditions on dengue transmission.

Risk factors for increased vector density (larvae/pupae abundance and productivity)	
	Source
Preadult & adult niche/habitat requirements: containers	

Water temperature	Barrera 2009

Water temperature Rainfall Container type; artificial: buckets, plastic sheets, pots Vegetation; trees, leaf litter, algae	Barrera et al. 2006

Container type; discarded: toys, auto parts, storage, covers, recreational, water storage, cleaning, ornamental, pans	Barrera et al. 2006

Container type; unattended/discarded: utensils, implements, plastic covers, plastic tools Location; large lots with abundance of trees	Barrera et al. 2006

Preadult & adult niche/habitat requirements: landscape elements	

Terrestrial environment: level of tree coverage; proximity & density level of host population; water temperature	Cox et al. 2007

Housing distribution: location: urban versus rural; relative population density; host movement patterns	Harrington et al. 2005

Preadult & adult niche/habitat requirements: underground aquatic habitats	

Septic tanks (STs): condition: uncovered, cracked, incomplete seal; location: suburban or rural	Barrera et al. 2008

ST environment: temperature; humidity; degree of solid waste; condition	Burke et al. 2010

ST maintenance level: lack of access to public sewage system; collection with rain water	MacKay et al. 2009

**Table 3:** Surveillance and intervention/control methods.

Method	Results	Source
*Entomologic surveillance*		
Pupae/demographic-survey for determination of dengue threshold	Sequential sampling; practical & reliable technique	Barrera 2006
Predictive computer models for validating dengue threshold; mosquito simulation model (CIMSiM), and dengue simulation model (DENSiM)	Sequential sampling programs (SSP) efficient method	Barrera 2009
Larval control programs	Cost-effective in conjunction with early warning systems	McConnell et al. 2003
*Epidemiologic surveillance*		
Surveillance system PRDH and Dengue Branch (CDC)	Successful in rapid implementation of education efforts for general public and medical community (1994; 1998)	Rigau-Pérez et al. 2002
Laboratory-based, enhanced dengue surveillance system (EDSS)	Allowed for a more accurate, population-based estimate of dengue incidence and severity, in conjunction with simplified case definitions for DHF based on WHO criteria	Ramos et al. 2009
*Child-focused intervention programs*		
Pilot child-focused community program	Limited results: did not impact larvae indices	Winch et al. 2002; Clark et al. 2004
*Community intervention programs*		
Community-based, health education campaign	Assessed barriers to prevention: differential gender-based concerns; differential knowledge level among infected; “invisibility” of dengue compared with chronic diseases; lack of acceptance of responsibility for dengue prevention	Pérez-Guerra 2009 et al.
Community-based, health education campaign	Assessed barriers to prevention: responsibility (self, other, government); “invisibility” of dengue compared with chronic diseases; misconceptions based on older education messages	Pérez-Guerra 2005 et al.
Population-specific (relief workers) utilization patterns of protective measures (repellant use) in real-world setting (after hurricane)	Zero of 204 workers had laboratory evidence of dengue 2 years after study. Personally protective behaviors effective	O'Leary et al. 2002
