# Supplementary material for: Interrelationship between Climatic, Ecologic, Social, and Cultural Determinants Affecting Dengue Emergence and Transmission in Puerto Rico and Their Implications for Zika Response
Source: J Trop Med. 2017 Jun 22;2017:8947067. doi: 10.1155/2017/8947067 (PMC5498925; doi:10.1155/2017/8947067)
Supplement: Supplementary file 1 — Appendix A: Summary of all studies included in the review. This table provides information about the study design, limitations, findings, and recommendations. Appendix B: Summary table of study results grouped by the category of research (social, cultural, and/or ecological). Appendix C: Quality assessment checklist with 49 questions developed and used in this review. Appendix D: Results of the quality assessment. Each study was reviewed for whether it satisfied each of Fink's criteria, examined in the form of 49 questions. Depending on the study design applicable questions were answered with Yes, No, or Not Applicable (NA). A YES answer received 1 point, a NO answer received zero points and criteria that were not applicable because of the study design were disregarded. After all questions were answered for each study, the percentage of satisfied Fink criteria was calculated (total number of satisfied criteria divided by total number of applicable criteria). Each study was then given an overall quality score represented by the quintile range of the percentage of applicable Fink criteria answered in the affirmative. [file 8947067.f1.docx]

SUPPLEMENTARY MATERIAL

APPENDIX A

**Supplementary Table 1**: Table of studies included

| Reference; Quality Score | Study  Design | Study population | Setting | Study  Period | Data Source | Reasons for Study | Key Findings | Study Limitations | Recommended dengue prevention/ control strategies |
| --- | --- | --- | --- | --- | --- | --- | --- | --- | --- |
| Barrera 2009; 5 | Observational (Ecological) | *Ae. aegypti* pupae | Playa-Playita  Coqui  Las Mareas  Sabana Llana  Villa Carolina  El Comandante | 2004-2008 | Pupal demographic surveys (10) | Explore methods for making pupal surveys more practical and reliable; assess two approaches for simplifying the assessment of dengue vector populations by means of pupal demographic surveys. | Simplified pupal surveys; useful method for validating entomological threshold for dengue transmission; positive relationship between mean pupae/person and proportion of infested households (*R*^2^ = .86; *p* < .01). | Addresses limitations:  Pupal demographic thresholds for *Ae. Aegypti* have not been validated (only for exploratory purposes); Study did not take into account effect of temperature, vector movement or herd immunity | Assessment of the ecology of Ae. aegypti larvae and pupae can help target environmental management and other control measures towards the most productive categories of breeding sites; the use of simplified survey methods to validate entomological thresholds for dengue transmission; method for targeted vector control |
| Barrera et al. 2006a; 4 | Observational  (Ecological) | *Ae. aegypti*  larvae | Salinas | May-July 2004 | Environmental data (types of containers; trees/ premise; water volume; water temperature) | Investigate impact of environmental conditions on larval and pupal abundance & productivity | Pos. association between pupal productivity and number trees per premise, water volume (*F*_7, 1197_ = 32.8; *p* < .01; *R*^2^ = .16); neg. association with water temperature and immatures (≤29°C; *p* < .01); pos. association between larvae and pupae abundance and unattended container with leaf litter or algae (rainfall) [*F*_4,1228_ = 20.7; *p* < .01].  Presence or absence of trees (in urban yards) is an important determinant of the spatial segregation of *Ae.aegypti* | Does not address limitations; variables other than those observed could have influenced results (organic materials in containers). | Vector control intervention that would eliminate most of Ae. aegypti productivity with social interventions aimed at source reduction & improved yard management, e.g. target unattended containers & remove shaded larger containers (in conjunction with larviciding and biocontrol); presence and abundance of trees; can be used to predict pupal productivity |
| Barrera et al. 2006b; 3 | Observational (Ecological) | *Ae. aegypti* pupae (female) | Playa-Playita (Salinas) | May-December 2004 | Pupal demographic surveys (2) | Compute appropriate sample size for determining dengue transmission threshold | Sampling of 25 premises in first survey and 125 determined that densities of female pupae were above the epidemic threshold [*W*=.89; *X*^2^ = 72; df=9; *p* < .01].  ; sequential sampling is reliable and practical method for reducing the number of samples required for determining transmission threshold. | Limitations addressed. Low generalizability:  Results limited to Salinas; has not been tested in other geographic areas | Sequential sampling technique is efficient method to determine dengue transmission threshold in different settings; epidemic thresholds could confer efficiency to surveillance and control programs; Importance of pupal/ demographic surveys for dengue control to increase efficiency of source reduction programs |
| Barrera et al. 2006c; 4 | Observational (Ecological) | *Ae. aegypti* pupae | Playa-Playita (Salinas) | May-July; Oct-Dec 2004 | Pupal demographic surveys (2) | Assess environmental conditions for abundance and productivity of pupae | Premises with more containers & accumulation of discarded containers in yards produce more mosquitoes; unattended rain-filled containers in yards in trees shade with rainfall through foliage and lower water temperature had most pupae | Does not address limitations; Pupal survey approach has not been validated elsewhere | Significant reduction in Ae. aegypti population could be achieved by residents' management of their backyards: remove discarded containers; place containers under roof or upside down; frequent renewal & cleaning of animal watering pans, draining plant pots |
| Barrera et al. 2008; 4 | Observational (Ecological) | *Ae aegypti* pupae; adults | Playa-Playita  (Salinas) | 2004;  Nov 2005-January 2006 | Pupal/ adult demographic surveys | Evaluate effectiveness of current vector control program | Pos relationship between dengue transmission and aquatic habitats (STs) in dry season (*X*^2^ = 19.9, *p* < .01); STs produced several times more *Ae. aegypti* pupae than surface containers; STs important habitat of *Ae. aegypti* in suburban and rural PR; vector control in surface containers may not be sufficient to prevent transmission | Does not address limitations; Barerra did not examine whether or not Ae. aegypti breeding was influenced by seasonal environmental conditions; selection bias | Repairing STs r replacing them with sewage system may reduce dengue burden in PR; 1) identify main types of containers producing Ae. aegypti; 2) evaluate efficiency of current control measures; 3) report presence of other mosquito species; 4) assess the state of all public services |
| Bennett et al. 2010; 4 | Observational (Ecological, modeling) | Human population; DEN-4 population | Multiple locations | 1981-1998 | Epidemiologic & sequence data; active surveillance data (PR DOH data base;); U.S. census data | Document the relationship between changes in pop size & structure and virus diversity | Direct relationship between changes in pop size & structure and virus diversity (*r* = .90, p<.01); human pop density associates with DEN-4; DHF | Limitations addressed: Possible flaws in epidemiologic surveillance data (oversampling during epidemic years) | More active surveillance of DENV activity to prevent missing dengue peaks & troughs. |
| Burke et al. 2010; 4 | Observational (Ecological) | *Ae aegypti* larvae/ adults; *Culex quinquefasciatus* larvae/ adults | Playa-Playita (Salinas) | Feb-Apr 2008 | Larvae/ adult septic tank surveys | Document relationship between *Ae. aegypti* larval presence, abundance & environment | Pos relationship between number of mosquitoes and condition of STs (*R*^2^ = .11). STs have the potential to maintain dengue transmission during the dry season. | Limitations addressed: Provided no evidence that larvae presence is linked to virus transmission; larvae only cited potential for dengue transmission | Increase efforts to convert homes from open sewage (ST) to sewer use to reduce potential for disease transmission |
| Clark et al. 2004; 2 | Qualitative | Participants in DOH public health programs on dengue (Elementary school, Head Start & Museum programs). | PR; elementary schools & museums | 1985-[2002] | Interviews/  observations | To assess knowledge and behaviors about dengue prevention among participants of national health programs | Community dengue prevention programs raised public awareness about dengue, transmission, and possible actions to prevent production of the mosquito vector in their back yards, and generated behavior change through social mobilization and communication, though they had limited impact on larval indices | Did not address limitations; No evidence on which sub-component (mass media; education in Elementary school; head start, children’s museum, boy’s scouts) had which effect. | Community members need to be equipped with necessary skills to keep containers free of larvae |
| Cox et al. 2007; 4 | Observational (Ecological) | *Ae. aegypti* and *Ae. mediovittatus* larvae | San Juan | Jul-Aug 2003 | Larvae demographic surveys | Investigate  habitat distribution of adult *Ae. aegypti* and *Ae. mediovittatus* | Pos association of *Ae. aegypti* with high-density housing, urban regions, and elevated water temperature in bamboo pots (*F* = 2.08; *p* < .05); *Ae, mediovittatus* was positively associated with forest areas, rural regions, and negatively correlated with water temperature;  mosquito species have specificity for landscape elements | Not addressed | Results contribute to identifying in which places (HDH, LDH, Forests) infections in humans might occur |
| Harrington et al. 2005; 4 | Observational  (Ecological) | *Ae. aegypti* adult | PR; Thailand | 1991-2001 | Adult demographic surveys | 1) Determine *Ae.aegypti* movement and dispersal patterns | *Ae. aegypti* do not disperse far from development site in and around home and tend to be clustered around households, in rural habitats and with abundance of human hosts & oviposition sites; suggests that people primary mode of dengue virus dissemination among and within communities | Limitations addressed: Study did not assess adverse effects of marking mosquitoes (with paint) on survival & dispersion. | Localized insecticide application around dengue patient's home not likely to prevent spread of dengue infection (this is because mosquitoes rather than people are most likely primary mode of dengue virus dissemination). |
| Johansson et al. 2009; 4 | Observational  (Ecological) | Dengue transmission in humans | PR | July 1986 – Dec 2006 | Suspected dengue infections PR Dept of Health & CDC&P | Examine relationship between climate & dengue incidence, statistically controlling for confounds (seasonal co-variation & spatial heterogeneity) | Positive association between monthly changes in temperature (*r* = .13 - .26) and precipitation (*r* = -.0006 - .0013) and monthly changes in dengue incidence by elevation. Spatial variations associated with differences in local climate; short-term association between weather variables & poverty index | Limitations addressed:  Does not address potential differences among DENV 1-4 | Climate is important factor in evaluating dengue prevention & control programs |
| Jury 2008; 4 | Observational  (Ecological) | Dengue transmission in humans | PR | 1979-2005 | Dengue Branch CDC and PR DOH | Examine relationship between dengue incidence & climate (seasonality; inter-annual variability) | Pos. pred. association between temperature & dengue incidence (*r* = .37, *p* < .05). | Limitations addressed: Study does not address confounding factors: endemic nature of dengue; mosquito control; public education. | Use climate forecasts to anticipate dengue epidemics and reduce adverse health impacts |
| Keating 2001; 4 | Observational  (Ecological, modeling) | PR | PR | 1988-1992 | NCDC/NOAA and Perez et al., 1994 | Test relationship between temperature fluctuations & dengue incidence | Pos. relationship b/w dengue incidence and temperature; monthly lagged temperature (*R*^2^ = .75). | Limitations addressed: Initial attempt at quantifying the relationship between dengue and temperature; (other factors need to be considered: precipitation; history of herd immunity; introduction of new serotype; demographic transitions). | Temperature models useful for forecasting cyclical dengue transmission |
| MacKay et al. 2009; 4 | Observational  (Ecological) | *Ae. aegypti* and *Cx. quinquefasciatus* larvae/ adults | Playa-Playita  Las Mareas  Sabana Llana | Nov 2006-Oct 2007 | Pupal and septic tank surveys | 1) Determine if *Ae.aegypti* uses STS throughout the year (not just in dry season)  2) Determine if STs produce more *Ae. aegypti* than surface containers  3) Test if *Ae aegypti* in STs are larger than usual. | STs produced large numbers of *Ae. aegypti* and *Cx. quinquefasciatus* throughout the year (*r*_s_ = .51-.84, *n* = 16, *p* < .05); no significant relationship with rainfall was found; STs produced more and larger *Ae. aegypti* than surface containers. | Not addressed: Surveyed STs were not randomly selected | Inspection & maintenance of STs to ensure that they are not serving as larval development sites for vector species, in communities lacking sewage systems |
| McConnell et al. 2003; 5 | Observational (Ecological) | PR | PR | 1982-1989 | Dengue Branch CDC and DOH dengue data base; U.S. census data | Provide estimates of cost-effectiveness of larval control programs; show that early warning systems can improve cost-effectiveness of larval control programs. | Emergency larval control programs are cost effective in conjunction with early warning systems in providing information on possible outbreak of dengue (US$ 4.50 per person, reducing dengue transmission by 50%). | Study used data from 1983-1989 and results underestimated cost-effectiveness of emergency larval control; study does not address interrelated factors that effect possible success of anti-larval program (such as herd immunity of a community, serotypes in circulation, climatic variables, community perceptions of larval control programs). | Provide incentives for the incorporation of early warning systems into larval control programs, even if they are not always 100% accurate, as they may have substantial benefit. |
| Mendez-Lazaro et al. 2014 | Observational (Ecological, modeling) | PR | San Juan | 1978-2012 | NOAA-National Climatic Data Center, National Oceanographic Data Center, Dengue Branch CDC and DOH | Assess the relationship between environmental parameters and the frequency of dengue | Association between. Rainfall, temperature and dengue incidence;  between 1992 and 2011, transmission increased by a factor of 3.4 for each 1 °C increase and this accelerate to 5.2 between 2007 to 2011 | Short-term variability difficult to assess in the context of longer-term trends Causality not explained | Need for inter-disciplinary collaboration. Results help understand possible impacts of different climate change scenarios in planning for social adaptation and interventions. |
| Morin et al 2015 | Observational (Ecological, modeling) | PR | San Juan | 2010-2013 | NOAA-National Climatic Data Center, Dengue Branch CDC and DOH | Examine how interactions among meteorological variables, vectors and the dengue  virus influence transmission | Rainfall  strongly modulates the timing of dengue (e.g., epidemics occurred earlier during rainy  years) while temperature modulates the annual number of dengue fever cases. | Some parameters based on few studies, serotype variation not accounted for, cases largely based on passive surveillance system | Meteorological factors have a time-variable influence on dengue transmission  relative to other important environmental and human factors. |
| O'Leary et al. 2002; 4 | Mixed Method | Relief workers in PR | PR; multiple locations | Oct-Nov 1998 | Outbreak investigation; patient questionnaires; blood specimens; Dengue Branch CDC and DOH dengue incidence data | Assess dengue risk in relief workers after Hurricane George and during 1998 dengue epidemic | Pre-travel messages contribute to protective behavior in travelers in endemic areas. | Relied on human recall of prevention strategies used | Educational messages for travelers to endemic areas should emphasize personal protective measures, including the use of insect repellents; importance of active surveillance (laboratory diagnosis). |
| Pérez-Guerra et al. 2005; 3 | Qualitative | Human participants | Arecibo  Patillas  San Juan  Villalba | Feb-May 2001 | Interviews; Dengue Branch CDC and DOH dengue data bases | Understand KAP related to dengue prevention and elicit ideas for future prevention campaigns | Barriers to community involvement in dengue prevention program:1) misconceptions about dengue; 2) "invisibility" of dengue; 3) responsibility. Dengue is not important to the general public in Puerto Rico | Sample biased towards those who do not move around (i.e., are stable) | Improve existing dengue prevention programs: 1) develop community groups to identify community priorities on prevention; 2) develop volunteer groups to deliver prevention messages; 3) make house visits to demonstrate specific control measures; 4) conduct complementary media campaign to support new strategies |
| Pérez-Guerra et al. 2009; 3 | Qualitative | 55 focus group participants | Carolina  Guaynabo  San Juan | Sept-Oct 2003 | Interviews | Explore differences in attitudes towards dengue and its prevention by gender & prior dengue infection; develop messages to promote *Ae. aegypti* control | Barriers to sustained dengue prevention included: 1) misconceptions from outdated educational material; 2) "invisibility" of dengue compared with chronic diseases; 3) lack of acceptance of responsibility for dengue prevention | Biased towards recall of limited number of participants. | Address structural problems that increase mosquito populations; improve access to information on garbage collection and water disposal; increase publicity and information about dengue; need to target men’s and women’s issues differently and directly. |
| Ramos et al. 2008; 4 | Observational  (Ecological) | Suspected dengue patients | Patillas | June 2005-May2006 | DOH serologic & virologic dengue data | Provide a more accurate estimate of incidence of symptomatic dengue infection | Enhanced surveillance useful for detecting symptomatic infections; simplified case definitions for severe dengue useful in clinic-based surveillance | Underestimates rates because of some dengue symptomatic people not seeking care | Clinic-based surveillance allows for more accurate population-based estimate of dengue incidence and measure of clinical severity of dengue infection |
| Ramos et al. 2009; 5 | Observational  (Ecological) | Laboratory-positive and –negative dengue patients | Patillas | June 2005- May 2006 | DOH serologic & virologic dengue data | Define early clinical features of dengue-infected children; define which clinical features predict laboratory-positive dengue infection | Early diagnosis useful in reducing dengue transmission in community, although laboratory confirmation still necessary for accurate diagnosis | Missing data; some tests indeterminate; possible influence by local & transient disease patterns | Identifying dengue early in clinical course could be useful in reducing virus transmission in community |
| Rigau-Pérez et al. 2002; 5 | Observational  (Ecological) | Laboratory-positive dengue patents | San Juan,  Ponce Univ Hosp | 1998-1999 | DOH serologic & virologic dengue data | Evaluate the clinical severity of DEN-1-4 infections in 1998 | 1998 DEN-3 infections could not have been detected without virologic surveillance | DEN-3 infection rate limited to reported cases only. | To minimize dengue mortality, vector control efforts needs to be ensured in conjunction with secondary prevention, such as an adequate medical care system (medical education, sufficient resources, appropriate treatment locations) |
| Rigau-Pérez et al. 2001; 4 | Observational  (Ecological) | PR | PR | 1995-1997 | PRDH & Dengue Branch, CDC&P | Highlight local conditions & epidemiologic history in determining dengue risk | No positive relationship between increase in dengue transmission & 1995 hurricane & 1996 floods; due to seasonal patterns; no correlation between increased transmission and rationed water supply and closed local landfills | Did not assess impact of climate on dengue pattern | Importance of laboratory-based dengue surveillance as long-term activity |
| Rigau-Pérez et al. 2001a; 4 | Observational  (Ecological) | PR | PR | 1994-1995 | Outbreak investigation;  DOH serologic & virologic dengue data | Describe 1994-1995 DHF epidemic and surveillance mechanisms used | No positive relationship between increase in dengue transmission & environmental changes (increased water storage due to rationing of water supply and illegal dumping due to closures of landfills) | Unable to assess relation between transmission & water rationing or landfill closure | Surveillance systems useful during epidemic to guide response |
| Winch et al. 2002; 3 | Mixed Method | PR children & adults | PR | Apr-Jul 1995 | Surveys | Assess impact of education campaigns on knowledge, behavior & infestation | Community programs showed significant positive impact on knowledge and behavior related to dengue prevention (*p* ranged from .038 - .001) | No pre-assessment of knowledge or behaviors | School programs need to increase parental involvement in dengue control; greater emphasis on the skills necessary for community members to keep containers free of mosquito larvae would increase program effectiveness |

APPENDIX B: Summary table of social, cultural, climatic and ecological research on dengue (categories adapted from Caprara et al. 2009; Arunachalam et al. 2010 & WHO 2011). Factors are italic; variables are in bold.

|  |  | Social research | | | | | Cultural research | | Climatic research | Ecological research | | |
| --- | --- | --- | --- | --- | --- | --- | --- | --- | --- | --- | --- | --- |
|  | Study first author; year; quality score | *Population growth* **(urbanization)** | *Population movement* **(migration, travel)** | *Household characteristics*  **(discarded & unattended containers, yard maintenance,**  **poverty index** | *Public infrastructure*  **(Sewage disposal; ST maintenance)** | *Public disease prevention & control programs*  **(Entomologic & epidemiologic disease surveillance)** | *Knowledge, attitude and practice (KAP)* | *Social organization*  (**Gender)** | *Climate* **(Precipitation, temperature, ENSO)** | *Landscape elements* **(HDH, LDH, Forests)** | *Pre-adult and adult niche and habitat requirements*  **(Container type, location, vegetation,**  **water, and temperature)** | *Vector density*  **Larval and pupae abundance and productivity** |
| 1 | Barrera 2009 |  |  |  |  | Vector Control |  |  | Temperature  (Positive predictive correlation b/w temperature & pupal counts) |  |  | Minimum number of mosquitoes/ person required for transmission (Entomologic transmission threshold) |
| 2 | Barrera et al. 2006a |  |  | Artificial surface container (buckets, plastic sheets, plant post); Trees; leaf litter, algae |  |  |  |  | Rain; water temperature |  | Outdoors (yard) w/ trees;  Urban | Vector quality (body mass of emerging females) |
| 3 | Barrera et al. 2006b |  |  | Discarded containers (toys, repairs, object storage, covers, recreational, water storage, cleaning, ornamental, drinking pans, d e.g. drums, flower vases, pots, cisterns) |  | Vector Control |  |  |  |  |  | Number of female pupae/ person (epidemic threshold) |
| 4 | Barrera et al. 2006c |  |  | Unattended discarded containers, utensils, implements (esp. plastic covers & plastic tools); premises w/ large lot sizes & abundance of trees |  |  |  |  | Rain; (water) temperature |  | Outdoors (yard) w/ trees;  urban s | Ae. aegypti productivity (pupae as proxy for # of adults) |
| 5 | Barrera et al. 2008 |  |  |  | Condition of septic tanks in suburban & rural area: open, broken, incompletely sealed |  |  |  |  | Dry season | Underground aquatic habitats (ST) | Number of pupae/person (threshold density) for dengue transmission |
| 6 | Bennett et al. 2010 | Urbanization; Population size; pop density |  |  |  |  |  |  | Dry season |  |  |  |
| 7 | Burke et al. 2010 |  |  |  | Cracking of tank wall or cover; uncovered openings |  |  |  | Dry season |  | Warm, moist environment of septic tanks containing raw sewage |  |
| 8 | Clark et al. 2004 |  |  |  |  |  | Inadequate public health education programs |  |  |  |  |  |
| 9 | Cox et al. 2007 | HDH in urban areas |  |  |  |  | Bamboo pots |  | Water temperature (sun) |  | Terrestrial environments w/ or w/t tree coverage & associated with humans; urban |  |
| 10 | Harrington et al. 2005 |  | Population movement in rural communities | House distribution & pattern in rural areas |  |  |  |  |  |  | Near houses w/ abundance of human hosts & oviposition sites; rural |  |
| 11 | Johansson et al. 2009 |  |  | Poverty Index |  |  |  |  | Rainfall, temperature |  |  |  |
| 12 | Jury 2008 |  |  |  |  |  |  |  | Rainfall, temperature, ENSO |  |  |  |
| 13 | Keating 2001 |  |  |  |  |  |  |  | Temperature |  |  |  |
| 14 | Mackay et al. 2009 |  |  |  |  | Septic tanks are unmaintained; lack of access to public sewage system; |  |  |  | Rain |  | Adult mosquito size |
| 15 | McConnell et al. 2003 |  |  |  |  | Surveillance (costs of larval control programs & early warning systems) | Knowledge of benefits of larval control programs; early warning systems |  |  |  |  |  |
| 16 | Mendz-Lazaro et al. 2014 |  |  |  |  |  |  |  | Sea surface temperature (positive association with increase in dengue incidence), rain |  |  |  |
|  |  |  |  |  |  |  |  |  |  |  |  |  |
| 17 | Morin et al. 2015 |  |  |  |  |  |  |  | Air temperature, daily total precipitation, latitude, and  container habitat area, height, and composition. |  |  | Simulated larval/pupal carrying capacity rates, adult daily survival rates, length of infectious period |
| 18 | O'Leary et al. 2002 |  | Dengue risks of relief workers |  |  | Inadequate dengue education & serodiagnostic services | Knowledge of prevention methods |  |  |  |  |  |
| 19 | Pérez-Guerra et al. 2005 |  |  |  |  | Inadequate community dengue prevention programs | Lack of perceived importance of dengue prevention |  |  |  |  |  |
| 20 | Pérez-Guerra et al. 2009 |  |  |  |  | Lack of health education programs |  |  |  |  |  |  |
| 21 | Ramos et al. 2008 |  |  |  |  | Benefits of epidemiological surveillance system |  |  |  |  |  |  |
| 22 | Ramos et al. 2009 |  |  |  |  | Benefits of current diagnostic systems |  |  |  |  |  |  |
| 23 | Rigau-Pérez et al. 2002 |  |  |  |  | Benefits of well established surveillance program & extensive virologic testing |  |  |  |  |  |  |
| 24 | Rigau-Pérez et al. 2001 |  |  | Rationed water supply; closed local landfills |  |  |  |  | Hurricane; floods |  |  |  |
| 25 | Rigau-Pérez et al. 2001a |  |  |  |  | Disease Surveillance: Epidemiological  (Active & Passive Surveillance)  Dengue surveillance & dengue diagnosis |  |  |  |  |  |  |
| 26 | Winch et al. 2002 |  |  |  |  | Dengue prevention programs | Impact on knowledge and behavior related to dengue prevention |  |  |  |  |  |

QUALITY ASSESSMENT CHECKLIST

*Adapted from* Fink, 2010

Design & Sampling Method

For Experiments

1. Is more than one group is included in the study, are the participants randomly assigned to each?
2. Are the participants measured over time and are the number of observations explained and justified?
3. If observations or measures are made over time, are the choice and effects of the time period explained?
4. Are any of the participants ‘blinded’ to the group to which they belong?
5. If historical controls are used are they explained and justified?
6. Are the effects of internal validity explained?
7. Are the effects of external validity explained?
8. If a sample is used are the subjects randomly selected?
9. Is the sample used relevant to the population of concern?
10. If a sample is selected with nonrandom method, is there evidence to suggest it is similar to the target population?
11. If groups are not equivalent at baseline is this problem addressed in the analysis?
12. Are inclusion criteria mentioned?
13. Are exclusion criteria mentioned?
14. Is the sample size justified?
15. Is information about the target population addressed?
16. If stratified sampling is used is it justified?
17. Is information on who from the target population is eligible to participate?
18. Are those eligible similar to those who agree on all factors?
19. Are those eligible similar to those who do not agree?
20. Is there information on those who are lost to follow-up or drop out?
21. Is the issue of any missing data addressed?

For Observational Studies, questions 1, 4 & 5 were not applicable.

For Cross-sectional Studies (aka Survey or Descriptive designs) and Qualitative Studies, questions 1 – 5 were not applicable.

Reliability And Validity Of Data Collection

1. Are all key variables defined?
2. Is information provided on the measure type, content, etc.?
3. Are any intervals between administrations (measurements) explained and justified?
4. Is the measure reliable?
5. Is the measure valid?

Program Or Intervention

1. Are the specific program objectives of the intervention described?
2. Is the make-up of the intervention and the control clearly described?
3. Were deviations in the intervention or control addressed?
4. Is there sufficient information on the settings?

Data Analysis

1. Are the research questions clearly stated?
2. Are the independent (predictor) variables defined?
3. Are the dependent (outcome) variables defined?
4. Are the statistical methods adequately described?
5. Is a reference provided for the statistical program used to analyze the data?
6. Are the statistical methods justified?
7. Is the purpose of the analysis clear?
8. Are potential confounds addressed?
9. Is statistical versus practical significance addressed?

Results

1. Are the study questions (hypotheses) clearly stated?
2. Are all study questions answered?
3. Are negative findings presented?
4. Are multiple comparisons explained?
5. Are response rates given for each group?
6. Are text and tables, figures and graphs consistent?

Conclusions

1. Are conclusions limited to the findings based on the sample, setting and program?
2. If findings are compared to other studies, is the equivalence of the study groups addressed (i.e., sample, setting and intervention)?
3. Are limitations of design, sampling, data collection, etc., described?
4. Are any limitations’ impact on confidence in conclusions addressed?

OVERALL QUALITY RATING (Percentage of Questions Answered)

0-19 = 1

20-39 = 2

40-59 = 3

60-79 = 4

80-100 = 5

APPENDIX D: Dengue studies quality assessment table

| **Citation** | **Column1** | **Column2** | **Column3** | **Column4** | **Column5** | **Design & Sampling Method** | | |
| --- | --- | --- | --- | --- | --- | --- | --- | --- |
|  | Q1 | Q2 | Q3 | Q4 | Q5 | Q6 | Q7 | Q8 |
| Barrera 2009 | NA | 1 | 1 | NA | NA | NA | NA | 1 |
| Barrera et al. 2006a | NA | 1 | 1 | NA | 1 | NA | NA | 1 |
| Barrera et al. 2006b | NA | 1 | 1 | NA | NA | NA | NA | 1 |
| Barrera et al. 2006c | NA | 1 | 1 | NA | NA | NA | NA | 1 |
| Barrera et al. 2008 | 0 | 1 | 1 | NA | 1 | NA | NA | 0 |
| Bennett et al. 2010 | NA | 1 | 1 | NA | NA | NA | NA | 1 |
| Burke et al. 2010 | NA | 1 | 1 | NA | NA | NA | NA | 0 |
| Clark et al. 2004 | NA | NA | NA | NA | NA | NA | NA | 0 |
| Cox et al. 2007 | NA | 1 | 1 | NA | NA | NA | NA | 0 |
| Harrington et al. 2005 | NA | 1 | 1 | NA | NA | NA | NA | 0 |
| Johansson et al. 2009 | NA | 1 | 1 | NA | NA | NA | NA | 0 |
| Jury 2008 | NA | 1 | 1 | NA | NA | NA | NA | NA |
| Keating 2001 | NA | 1 | 1 | NA | NA | NA | NA | 0 |
| Mackay et al. 2009 | NA | 1 | 1 | NA | NA | NA | NA | 0 |
| McConnell et al. 2003 | NA | NA | NA | NA | NA | NA | NA | NA |
| Mendez-Lazaro et al 2014 | NA | 1 | 1 | NA | NA | NA | NA | NA |
| Morin et al. 2015 | NA | NA | NA | NA | NA | NA | NA | 0 |
| O'Leary et al. 2002 | NA | NA | NA | NA | NA | NA | NA | 0 |
| Pérez-Guerra et al. 2005 | NA | NA | NA | NA | NA | NA | NA | 0 |
| Pérez-Guerra et al. 2009 | NA | NA | NA | NA | NA | NA | NA | 0 |
| Ramos et al. 2008 | NA | 1 | 1 | NA | NA | NA | NA | 0 |
| Ramos et al. 2009 | NA | 1 | 1 | NA | NA | NA | NA | NA |
| Rigau-Pérez et al. 2002 | NA | 1 | 1 | NA | NA | NA | NA | NA |
| Rigau-Pérez et al. 2001 | NA | 1 | 1 | NA | NA | NA | NA | NA |
| Rigau-Pérez et al. 2001a | NA | 1 | 1 | NA | NA | NA | NA | NA |
| Winch et al. 2002 | 0 | 0 | 1 | 0 | NA | NA | NA | 0 |

| **Citation** | **Column8** | **Column9** | **Column10** | **Column11** | **Column12** | **Column13** | **Column14** | **Column15** | **Column16** |
| --- | --- | --- | --- | --- | --- | --- | --- | --- | --- |
|  | Q9 | Q10 | Q11 | Q12 | Q13 | Q14 | Q15 | Q16 | Q17 |
| Barrera 2009 | 1 | NA | NA | 1 | 0 | 1 | 1 | NA | NA |
| Barrera et al. 2006a | 1 | NA | NA | 1 | 0 | 0 | 1 | NA | NA |
| Barrera et al. 2006b | 1 | NA | NA | 0 | 0 | NA | 0 | NA | NA |
| Barrera et al. 2006c | 1 | NA | NA | 0 | 0 | NA | 1 | NA | NA |
| Barrera et al. 2008 | 1 | 1 | NA | 0 | 0 | 0 | 1 | NA | NA |
| Bennett et al. 2010 | 1 | NA | NA | 0 | 0 | 1 | 1 | NA | NA |
| Burke et al. 2010 | 1 | 1 | 0 | 1 | 1 | 0 | 1 | NA | NA |
| Clark et al. 2004 | 1 | NA | NA | NA | NA | NA | 1 | NA | NA |
| Cox et al. 2007 | 1 | 0 | NA | 1 | 0 | 0 | 1 | NA | NA |
| Harrington et al. 2005 | 1 | 0 | 0 | 1 | 1 | 0 | 1 | NA | NA |
| Johansson et al. 2009 | 1 | 0 | 0 | 1 | 1 | 1 | 1 | NA | NA |
| Jury 2008 | NA | NA | NA | NA | NA | NA | NA | NA | NA |
| Keating 2001 | 1 | 0 | 0 | 1 | 0 | 0 | 1 | NA | NA |
| Mackay et al. 2009 | 1 | 0 | NA | 1 | 0 | 0 | 1 | NA | NA |
| McConnell et al. 2003 | NA | NA | NA | NA | NA | NA | 1 | NA | NA |
| Mendez-Lazaro et al. 2014 | NA | NA | NA | NA | NA | NA | NA | NA | NA |
| Morin et al. 2015 | NA | NA | NA | NA | NA | NA | NA | NA | NA |
| O'Leary et al. 2002 | 1 | 0 | NA | 1 | 0 | 0 | 1 | NA | NA |
| Pérez-Guerra et al. 2005 | 0 | 0 | NA | 1 | 1 | 0 | 0 | NA | NA |
| Pérez-Guerra et al. 2009 | 0 | 0 | NA | 1 | 1 | 0 | 1 | NA | NA |
| Ramos et al. 2008 | 0 | 0 | NA | 1 | 1 | 0 | 1 | NA | NA |
| Ramos et al. 2009 | 1 | NA | NA | 1 | 1 | NA | 1 | NA | NA |
| Rigau-Pérez et al. 2002 | 1 | NA | NA | 1 | NA | NA | NA | NA | NA |
| Rigau-Pérez et al. 2001 | 1 | NA | NA | 1 | NA | NA | 1 | NA | NA |
| Rigau-Pérez et al. 2001a | 1 | 1 | NA | 1 | NA | NA | 1 | NA | NA |
| Winch et al. 2002 | 1 | 0 | NA | 1 | 0 | 0 | 1 | NA | NA |

| **Citation** | **Column17** | **Column18** | **Column19** | **Column20** | **Column21** | **Column22** | **Data Collection** | **Column23** |
| --- | --- | --- | --- | --- | --- | --- | --- | --- |
|  | Q18 | Q19 | Q20 | Q21 | Q22 | Q23 | Q24 | Q25 |
| Barrera 2009 | NA | NA | NA | 1 | 1 | 1 | NA | 1 |
| Barrera et al. 2006a | NA | NA | NA | 0 | 1 | 1 | NA | 1 |
| Barrera et al. 2006b | NA | NA | NA | 0 | 1 | 1 | 1 | 1 |
| Barrera et al. 2006c | NA | NA | NA | 0 | 1 | 1 | NA | 1 |
| Barrera et al. 2008 | NA | NA | NA | 0 | 1 | 1 | 1 | 1 |
| Bennett et al. 2010 | NA | NA | NA | 0 | 1 | 1 | 1 | 1 |
| Burke et al. 2010 | NA | NA | NA | 0 | 1 | 1 | NA | 1 |
| Clark et al. 2004 | NA | NA | NA | NA | 1 | 0 | NA | NA |
| Cox et al. 2007 | NA | NA | NA | 0 | 1 | 1 | NA | 1 |
| Harrington et al. 2005 | NA | NA | NA | 0 | 1 | 1 | 1 | 1 |
| Johansson et al. 2009 | NA | NA | NA | 0 | 1 | 1 | 1 | 1 |
| Jury 2008 | NA | NA | NA | NA | 1 | 1 | NA | 1 |
| Keating 2001 | NA | NA | NA | 0 | 1 | 1 | NA | 1 |
| Mackay et al. 2009 | NA | NA | NA | 0 | 1 | 1 | NA | 1 |
| McConnell et al. 2003 | NA | NA | NA | 0 | 1 | 1 | NA | 1 |
| Mendez-Lazaro et al 2014 | NA | NA | NA | NA | 1 | 1 | NA | 1 |
| Morin et al. 2015 | NA | NA | NA | NA | 1 | 1 | NA | 1 |
| O'Leary et al. 2002 | NA | NA | 0 | 0 | 1 | 1 | NA | 1 |
| Pérez-Guerra et al. 2005 | NA | 0 | 1 | 0 | 1 | 1 | NA | 0 |
| Pérez-Guerra et al. 2009 | NA | 0 | 0 | 0 | 1 | 1 | 0 | 0 |
| Ramos et al. 2008 | NA | NA | NA | NA | 1 | 1 | NA | 1 |
| Ramos et al. 2009 | NA | NA | NA | 1 | 1 | 1 | NA | 1 |
| Rigau-Pérez et al. 2002 | NA | NA | NA | NA | 1 | 1 | NA | 1 |
| Rigau-Pérez et al. 2001 | NA | NA | NA | NA | 1 | 1 | NA | 1 |
| Rigau-Pérez et al. 2001a | NA | NA | NA | NA | 1 | 1 | NA | 1 |
| Winch et al. 2002 | 0 | NA | 0 | 0 | 1 | 1 | 1 | 1 |

| **Citation** | **Column24** | **Column25** | **Program** | **Column26** | **Column27** | **Column28** | **Column29** | **Column30** | **Column31** |
| --- | --- | --- | --- | --- | --- | --- | --- | --- | --- |
|  | Q26 | Q27 | Q28 | Q29 | Q30 | Q31 | Q32 | Q33 | Q34 |
| Barrera 2009 | NA | NA | NA | NA | NA | 1 | 1 | 1 | 1 |
| Barrera et al. 2006a | 1 | NA | NA | NA | NA | 1 | 1 | 1 | 1 |
| Barrera et al. 2006b | 1 | NA | NA | NA | NA | 1 | 1 | 1 | 1 |
| Barrera et al. 2006c | 1 | NA | NA | NA | NA | 1 | 1 | 1 | 1 |
| Barrera et al. 2008 | 1 | 1 | 1 | 0 | 1 | 1 | 1 | 1 | 1 |
| Bennett et al. 2010 | 1 | NA | NA | NA | NA | 1 | 1 | 1 | 1 |
| Burke et al. 2010 | 1 | NA | NA | NA | NA | 1 | 1 | 1 | 1 |
| Clark et al. 2004 | NA | 1 | 1 | 0 | 1 | NA | NA | NA | NA |
| Cox et al. 2007 | 1 | NA | NA | NA | NA | 1 | 1 | 1 | 1 |
| Harrington et al. 2005 | 1 | NA | NA | NA | NA | 1 | 1 | 1 | 1 |
| Johansson et al. 2009 | 1 | NA | NA | NA | NA | 1 | 1 | 1 | 1 |
| Jury 2008 | 1 | NA | NA | NA | NA | 1 | 1 | 1 | 1 |
| Keating 2001 | 1 | NA | NA | NA | NA | 1 | 1 | 1 | 1 |
| Mackay et al. 2009 | 1 | NA | NA | NA | NA | 1 | 1 | 1 | 1 |
| McConnell et al. 2003 | 1 | 1 | 1 | 0 | 1 | 1 | 1 | 1 | 1 |
| Mendez-Lazaro et al 2014 | 1 | NA | NA | NA | NA | 1 | 1 | 1 | 1 |
| Morin et al. 2015 | 1 | NA | NA | NA | NA | 1 | 1 | 1 | 1 |
| O'Leary et al. 2002 | 1 | NA | NA | NA | NA | 1 | 1 | 1 | 1 |
| Pérez-Guerra et al. 2005 | 0 | NA | NA | NA | NA | 1 | 0 | 0 | NA |
| Pérez-Guerra et al. 2009 | 0 | NA | NA | NA | NA | 1 | 0 | 0 | NA |
| Ramos et al. 2008 | 1 | NA | NA | NA | NA | 1 | 1 | 1 | 1 |
| Ramos et al. 2009 | 1 | NA | NA | NA | NA | 1 | 1 | 1 | 1 |
| Rigau-Pérez et al. 2002 | 1 | 1 | NA | NA | 1 | 1 | 1 | 1 | 1 |
| Rigau-Pérez et al. 2001 | 1 | NA | NA | NA | NA | 1 | NA | NA | 1 |
| Rigau-Pérez et al. 2001a | 1 | NA | NA | NA | NA | 1 | NA | NA | 1 |
| Winch et al. 2002 | 1 | 1 | 1 | 0 | 1 | 1 | 1 | 1 | 1 |

| **Citation** | **Column32** | **Data Analysis** | **Column33** | **Column34** | **Column35** | **Column36** | **Column37** | **Results** | **Column38** |
| --- | --- | --- | --- | --- | --- | --- | --- | --- | --- |
|  | Q35 | Q36 | Q37 | Q38 | Q39 | Q40 | Q41 | Q42 | Q43 |
| Barrera 2009 | 1 | 1 | 1 | 0 | 0 | 1 | 1 | 0 | 1 |
| Barrera et al. 2006a | 1 | 1 | 1 | 0 | 0 | 1 | 1 | 0 | 1 |
| Barrera et al. 2006b | 1 | 1 | 1 | 0 | 0 | 1 | 1 | 0 | 1 |
| Barrera et al. 2006c | 1 | 1 | 1 | 0 | 0 | 1 | 1 | 0 | 1 |
| Barrera et al. 2008 | 1 | 1 | 1 | 0 | 0 | 1 | 1 | 0 | 1 |
| Bennett et al. 2010 | 1 | 1 | 1 | 1 | 1 | 1 | 1 | 0 | 1 |
| Burke et al. 2010 | 1 | 1 | 1 | 0 | 0 | 1 | 1 | 1 | 1 |
| Clark et al. 2004 | NA | NA | NA | NA | NA | 0 | 0 | 0 | NA |
| Cox et al. 2007 | 1 | 1 | 1 | 0 | 0 | 1 | 1 | 1 | 1 |
| Harrington et al. 2005 | 1 | 1 | 1 | 0 | 0 | 1 | 1 | 0 | 1 |
| Johansson et al. 2009 | 1 | 1 | 1 | 1 | 0 | 1 | 1 | 1 | 1 |
| Jury 2008 | 1 | 1 | 1 | 0 | 0 | 1 | 1 | 1 | 1 |
| Keating 2001 | 1 | 1 | 1 | 1 | 0 | 1 | 1 | 1 | 1 |
| Mackay et al. 2009 | 1 | 1 | 1 | 0 | 0 | 1 | 1 | 1 | 1 |
| McConnell et al. 2003 | 1 | 1 | 1 | 0 | 0 | 1 | 1 | 1 | 1 |
| Mendez-Lazaro et al. 2014 | 1 | 1 | 1 | NA | 0 | 1 | 1 | 0 | 0 |
| Morin et al. 2015 | 1 | 1 | 1 | NA | 1 | 1 | 1 | 0 | 1 |
| O'Leary et al. 2002 | 1 | 1 | 1 | 0 | 0 | 1 | 1 | 0 | NA |
| Pérez-Guerra et al. 2005 | NA | NA | 1 | 0 | NA | 1 | 1 | 0 | 1 |
| Pérez-Guerra et al. 2009 | NA | NA | 1 | 0 | NA | 1 | 1 | 0 | NA |
| Ramos et al. 2008 | 1 | 1 | 1 | 0 | 1 | 1 | 1 | 1 | NA |
| Ramos et al. 2009 | 1 | 1 | 1 | 0 | 0 | 1 | 1 | 1 | NA |
| Rigau-Pérez et al. 2002 | 1 | 1 | 1 | 0 | 0 | 1 | 1 | NA | NA |
| Rigau-Pérez et al. 2001 | 1 | 1 | 1 | 0 | 0 | 1 | 1 | NA | NA |
| Rigau-Pérez et al. 2001a | 1 | 1 | 1 | 0 | 0 | 1 | 1 | NA | NA |
| Winch et al. 2002 | 1 | 1 | 1 | 0 | 0 | 1 | 1 | 0 | 1 |

| **Citation** | **Column39** | **Column40** | **Column41** | **Conclusions** | **Column42** | **Column43** | **Column44** | **Column45** |
| --- | --- | --- | --- | --- | --- | --- | --- | --- |
|  | Q44 | Q45 | Q46 | Q47 | Q48 | Q49 | Pt Earned | Total Possible |
| Barrera 2009 | 1 | 1 | 1 | 0 | 1 | 1 | 27 | 31 |
| Barrera et al. 2006a | 0 | 1 | 1 | 0 | 0 | 0 | 24 | 33 |
| Barrera et al. 2006b | 0 | 1 | 1 | 0 | 0 | 0 | 21 | 32 |
| Barrera et al. 2006c | 1 | 1 | 1 | 1 | 0 | 0 | 23 | 31 |
| Barrera et al. 2008 | 0 | 1 | 1 | 1 | 0 | 0 | 29 | 42 |
| Bennett et al. 2010 | 0 | 1 | 1 | 1 | 0 | 0 | 29 | 35 |
| Burke et al. 2010 | 1 | 1 | 1 | NA | 0 | 0 | 25 | 33 |
| Clark et al. 2004 | NA | NA | 0 | 0 | 0 | 0 | 6 | 16 |
| Cox et al. 2007 | 0 | 1 | 1 | 1 | 0 | 0 | 23 | 33 |
| Harrington et al. 2005 | 0 | 1 | 1 | 0 | 1 | 0 | 24 | 35 |
| Johansson et al. 2009 | 0 | 1 | 1 | 1 | 1 | 1 | 29 | 35 |
| Jury 2008 | NA | 1 | 1 | 1 | 0 | 0 | 20 | 24 |
| Keating 2001 | NA | 1 | 1 | NA | 1 | 1 | 25 | 32 |
| Mackay et al. 2009 | NA | 1 | 1 | 0 | 0 | 0 | 22 | 32 |
| McConnell et al. 2003 | NA | 1 | 1 | 1 | 1 | 1 | 24 | 28 |
| Mendez-Lazaro et al. 2014 | NA | 1 | 1 | 0 | 0 | 0 | 16 | 23 |
| Morin et al. 2015 | NA | 1 | 1 | 0 | 1 | 1 | 20 | 23 |
| O'Leary et al. 2002 | 1 | 1 | 1 | 1 | 0 | 0 | 20 | 31 |
| Pérez-Guerra et al. 2005 | 1 | NA | 1 | 0 | 1 | 0 | 13 | 28 |
| Pérez-Guerra et al. 2009 | 1 | NA | 1 | 1 | 0 | 0 | 12 | 28 |
| Ramos et al. 2008 | NA | 1 | 1 | 1 | 1 | 1 | 25 | 30 |
| Ramos et al. 2009 | NA | 1 | 1 | 1 | 1 | 1 | 26 | 28 |
| Rigau-Pérez et al. 2002 | NA | 1 | 1 | 1 | 1 | 1 | 24 | 26 |
| Rigau-Pérez et al. 2001 | NA | 1 | 1 | 1 | 0 | 0 | 19 | 23 |
| Rigau-Pérez et al. 2001a | NA | 1 | 1 | 0 | 0 | 0 | 19 | 24 |
| Winch et al. 2002 | 0 | 1 | 1 | NA | 1 | 0 | 25 | 41 |

| **Citation** | ***Column46*** | **Column47** |
| --- | --- | --- |
|  | *Score* | Quality Rating |
| Barrera 2009 | 87% | 5 |
| Barrera et al. 2006a | 73% | 4 |
| Barrera et al. 2006b | 66% | 4 |
| Barrera et al. 2006c | 74% | 4 |
| Barrera et al. 2008 | 69% | 4 |
| Bennett et al. 2010 | 83% | 5 |
| Burke et al. 2010 | 77% | 4 |
| Clark et al. 2004 | 38% | 2 |
| Cox et al. 2007 | 69% | 4 |
| Harrington et al. 2005 | 69% | 4 |
| Johansson et al. 2009 | 83% | 5 |
| Jury 2008 | 83% | 5 |
| Keating 2001 | 78% | 4 |
| Mackay et al. 2009 | 69% | 4 |
| McConnell et al. 2003 | 86% | 5 |
| Mendez-Lazaro et al. 2014 | 70% | 4 |
| Morin et al. 2015 | 87% | 5 |
| O'Leary et al. 2002 | 65% | 4 |
| Pérez-Guerra et al. 2005 | 46% | 3 |
| Pérez-Guerra et al. 2009 | 43% | 3 |
| Ramos et al. 2008 | 83% | 5 |
| Ramos et al. 2009 | 93% | 5 |
| Rigau-Pérez et al. 2002 | 92% | 5 |
| Rigau-Pérez et al. 2001 | 83% | 5 |
| Rigau-Pérez et al. 2001a | 79% | 4 |
| Winch et al. 2002 | 61% | 4 |
